# GATA3 expression correlates with poor prognosis and tumor-associated macrophage infiltration in peripheral T cell lymphoma

**DOI:** 10.18632/oncotarget.11673

**Published:** 2016-08-29

**Authors:** Wei Zhang, Zi Wang, Yunping Luo, Dingrong Zhong, Yufeng Luo, Daobin Zhou

**Affiliations:** ^1^ Department of Hematology, Peking Union Medical College (PUMC) Hospital, Chinese Academy of Medical Sciences and PUMC, Beijing, 100730, China; ^2^ Department of Immunology, Institute of Basic Medical Science, Chinese Academy of Medical Science and Peking Union Medical College, Beijing, 100730, China; ^3^ Department of Pathology, PUMC Hospital, Chinese Academy of Medical Sciences and PUMC, Beijing, 100730, China

**Keywords:** GATA3, T-bet, peripheral T-cell lymphoma, tumor-associated macrophages, Hut78

## Abstract

Peripheral T cell lymphoma (PTCL) is an aggressive form of non-Hodgkin's lymphoma characterized by a poor prognosis. In this study, we examined the prognostic value of two T-cell-specific transcription factors, GATA3 and T-bet, in PTCL, uncovered the pathogenesis of PTCL, and investigated new PTCL therapeutic targets. Samples from 109 PTCL patients were examined for expression of GATA3, T-bet and CD68. High GATA3 expression correlated with poor survival in PTCL patients and with tumor-associated macrophage (TAM) infiltration, as indicated by the presence of CD68-positive cells. Multivariate analysis further confirmed that high GATA3 expression and Eastern Cooperative Oncology Group (ECOG) scores higher than 2 were independent predictors of patient survival. Using lentiviral transfection to induce stable *GATA3* knockdown in a PTCL cell line, we observed that GATA-3 knockdown in Hut78 cells decreased levels of *IL4*, *IL5*, *IL13* and *VEGF* mRNA and reduced the number of co-cultured U937 cells that differentiated towards the M2 phenotype. These results suggest that high GATA3 expression is a predictor of a poor prognosis in PTCL, and that T lymphoma cells promote M2-type macrophage differentiation through a GATA3-dependent mechanism.

## INTRODUCTION

Peripheral T cell lymphoma (PTCL), which is common in Asia [1], is an aggressive form of lymphoma associated with poor patient outcomes. Clinical hematologists have widely adopted the 2010 World Health Organization (WHO) criteria to classify PTCLs into various subtypes, with PTCL-not otherwise specified (PTCL-NOS) being the most common [2, 3]. PTCL-NOS is an exclusive diagnosis comprised of PTCL cases not classifiable as any defined T-cell lymphoma entity. The normal cellular components of PTCL-NOS have not been accurately identified, and the immunophenotypic profiles of PTCL-NOS are heterogeneous [4]. As a result, there is no consolidated chemotherapy regimen for PTCL-NOS, and patient survival rates are less than 30%. Thus, there is an urgent need to further improve PTCL classification and develop targeted therapies based on the signaling pathways that are aberrantly expressed in PTCL subtypes.

In T cells, the transcription factors GATA-binding protein 3 (GATA3) and T-box family transcription factor (T-bet) are T_H_2 and T_H_1 cell differentiation markers, respectively. GATA3 and T-bet antagonize each other to repress alternate cell fates. Large gene expression profiling performed on 121 PTCL-NOS cases found that T-bet-positive PTCL has a more favorable prognosis when compared with GATA3-positive PTCL [5]. The function of GATA3 has been previously reported in breast cancer tumorigenesis, but there are few reports of a link between T-bet and cancer formation.

As important constituents of the tumor microenvironment, tumor-associated macrophages (TAMs) are associated with malignant behaviors [6]. TAMs have been widely investigated in a variety of solid tumors, and there is a clear correlation between increased TAM content and poor survival [7, 8]. TAMs can be further classified into M1 and M2 types, with the M2 type promoting tumor cell proliferation, survival, infiltration, and metastasis, thus transforming the tumor microenvironment in an unfavorable manner. Interestingly, IL-10 may promote M2 polarization and maximize malignant tumor behavior [9]. In the present study, we analyzed the value of GATA3, T-bet, and CD68 expression as prognostic indicators in PTCL cases and explored the role of GATA3 in promoting macrophage differentiation utilizing an *in vitro* co-culture system.

## RESULTS

### Patient clinical characteristics

Clinical characteristics of patients in various subgroups are summarized in Table 1. The median age of all the evaluated patients was 46 years (range, 13–79 years). A male predominance was noted, with a male-to-female ratio of 2.2:1. Of the 109 patients, 73.4% were diagnosed with a stage III-IV tumor. Forty patients fell into the low-risk group (International Prognostic Index [IPI] scores of 0–2), and 65 patients fell into the high-risk group (IPI scores of 3–5).

**Table 1:** Clinical characteristics of the major subtypes of 109 PTCL samples

Characteristics	PTCL-NOS	NKT	AITL	ALCL		Total
				ALK+	ALK−	
Age > 60	20/60 (33.3)	2/18 (11.1)	6/11 (54.5)	0/4 (0.0)	1/4 (25.0)	29/109 (26.6)
Gender, male	38/60 (63.3)	13/18 (72.2)	9/11 (81.8)	3/4 (75.0)	3/4 (75.0)	75/109 (68.8)
B symptoms	47/58 (81.0)	16/18 (88.9)	11/11 (100.0)	4/4 (100.0)	2/4 (50.0)	90/107 (84.1)
LDH > normal	36/58 (62.1)	10/17 (58.8)	10/11 (90.9)	4/4 (100.0)	2/4 (50.0)	68/105 (64.8)
Ann Arbor stage III/IV	52/58 (89.7)	15/16 (93.8)	11/11 (100.0)	4/4 (100.0)	3/4 (75.0)	80/109 (73.4)
ECOG > 1	30/58 (51.7)	4/17 (23.5)	5/11 (45.5)	3/4 (75.0)	1/4 (25.0)	50/106 (47.2)
> 1 extranodal site	29/57 (50.9)	11/17 (64.7)	4/11 (36.4)	4/4 (100.0)	2/4 (50.0)	57/104 (54.8)
BM involvement	16/55 (29.1)	3/17 (17.6)	1/11 (9.1)	1/4 (25.0)	1/4 (25.0)	25/101 (24.8)
IPI > 2	35/57 (61.4)	10/18 (55.6)	7/11 (63.6)	4/4 (100.0)	3/4 (75.0)	65/105 (61.9)
Treatment with CHOP	26/60 (43.3)	3/18 (16.7)	5/11 (45.5)	3/4 (75.0)	1/4 (25.0)	42/109 (38.5)

CHOP, cyclophosphamide, vindesine, doxorubicin, prednisone; ECOG, Eastern Cooperative Oncology Group; LDH, lactate dehydrogenase.

All patients received 4 cycles of standard induction chemotherapy, followed by consolidation chemotherapy or autologous peripheral blood stem cell transplant if complete remission was achieved. Regimens included CHOP (cyclophosphamide + doxorubicin + vindesine + prednisone), hyperCVAD (Course A: cyclophosphamide +vindesine +doxorubicin +dexamethasone; Course B: methotrexate + cytosine arabinoside) and GDP-ML (gemcitabine +cisplatin +dexamethasone +methotrexate +Pegaspargase). The median OS was 380 days (95% CI, 233–526). The 1-year, 2-year, and 3-year OS rates were 58.7%, 30.4% and 25.1%, respectively.

### Master regulators of T_H_1 (T-bet) and T_H_2 (GATA3) cells are expressed in both PTCL primary samples and cell lines

**Figure 1:**
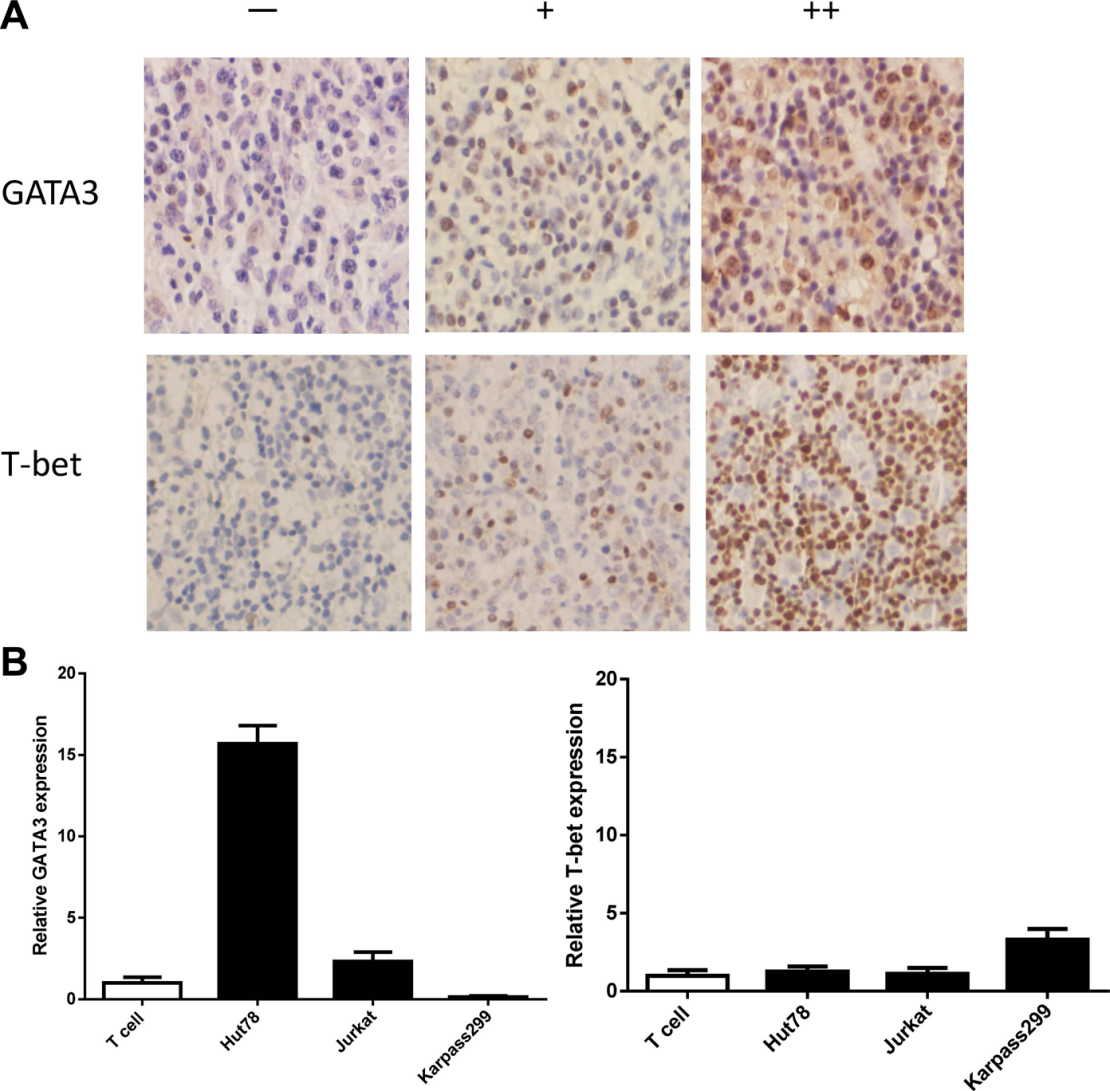
Expression of T-bet and GATA3 in clinical samples and PTCL cell lines (**A**) Representative PTCL cases of negative (−), positive Grade 1 (+) and positive Grade 2 (++) are shown. (**B**) Quantitative RT-PCR showed overexpression of GATA3 in Hut78 cells in comparison with T cells from healthy donors. For T-bet expression, there was a slight increase in Karpas 299 cells only.

Using immunohistochemistry we analyzed PTCL tissues for the expression and location of T-bet and GATA3. Expression of both T-bet and GATA3 was confined to nuclei (Figure 1A), in accordance with their roles as transcription factors. Quantitative RT-PCR showed overexpression of only GATA3 in Hut78 cells in comparison with T cells from healthy donors. For T-bet expression, only a slight increase was observed in Karpas 299 cells (Figure 1B).

Next, the expression of T-bet and GATA3 was detected in all PTCL subsets (Table 2), with total positive rates of 42.2% and 45.9%, respectively. Because of the limited sample size, further grading of the staining was only completed in the 5 major subtypes. The proportion of moderate immunostaining (10%–30%) for T-bet and GATA3 was as follows: 35.0% and 35.0% in PTCL-NOS, 22.2% and 50.0% in NKT, 27.3% and 54.5% in AITL, 50.0% and 50.0% in ALK+ ALCL, and 25.0 and 25.0% in ALK- ALCL. Strong staining (> 30%) for T-bet and GATA3 was observed as follows: 8.3% and 6.7% in PTCL-NOS, 22.2% and 5.6% in NKT, 0.0% and 18.2% in AITL, 0.0% and 50.0% in ALK+ ALCL, and 25.0% and 0.0% in ALK- ALCL.

**Table 2:** Expression of T-bet and GATA3 in 109 PTCL tumor samples by immunohistochemistry

	T-bet				GATA-3			
	No.	–	+	++	No.	–	+	++
PTCL-NOS	60	34 (56.7)	21 (35.0)	5 (8.3)	60	35 (58.3)	21 (35.0)	4 (6.7)
NKT	18	10 (55.6)	4 (22.2)	4 (22.2)	18	8 (44.4)	9 (50.0)	1 (5.6)
AITL	11	8 (72.7)	3 (27.3)	0 (0.0)	11	3 (27.3)	6 (54.5)	2 (18.2)
ALK+ ALCL	4	2 (50.0)	2 (50.0)	0 (0.0)	4	0 (0.0)	2 (50.0)	2 (50.0)
ALK- ALCL	4	2 (50.0)	1 (25.0)	1 (25.0)	4	3 (75.0)	1 (25.0)	0 (0.0)
Others	12	7 (58.3)	4 (33.3)	1 (8.3)	12	10 (83.3)	1 (8.3)	1 (8.3)
Total	109	63 (57.8)	35 (32.1)	11 (10.1)	109	59 (54.1)	40 (36.7)	10 (9.2)

### Correlation of T-bet and GATA3 expression with overall survival (OS)

**Figure 2:**
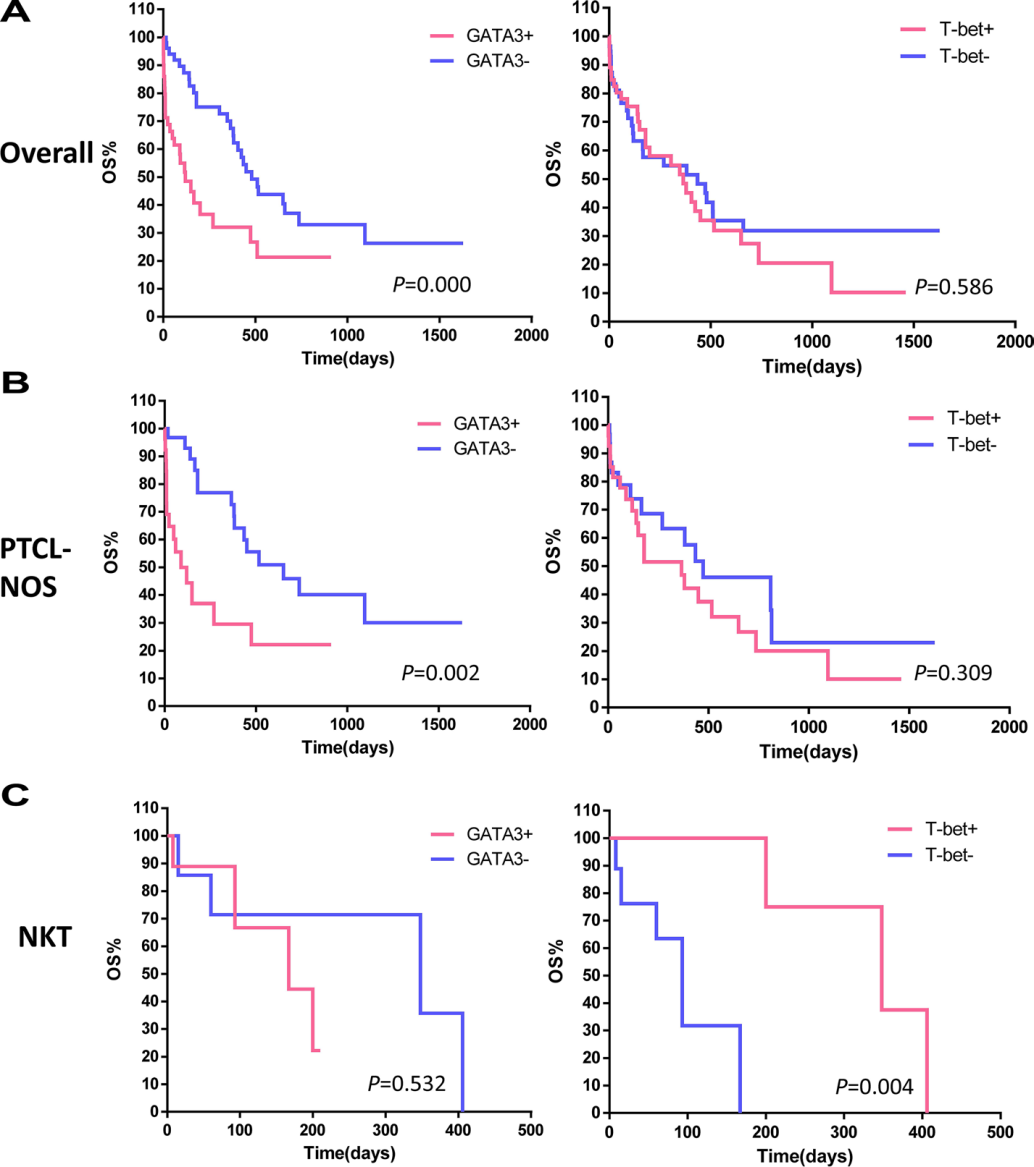
Kaplan-Meier survival analysis of PTCL subgroups based on T-bet and GATA3 expression (**A**) In the 109 patient cohort, GATA3 expression was associated with decreased overall survival (OS; log-rank, *p* = 0.000). T-bet expression was not correlated with OS. (**B**) In the PTCL-NOS subgroup, GATA3 expression identified a subset with inferior survival (log-rank, *p* = 0.002), while the stratification based on T-bet expression did not provide differences. (**C**) In the NKT lymphoma subgroup, GATA3 failed to identify a specific prognostic subgroup, while T-bet identified a subset with superior survival (log-rank, *p* = 0.004).

To estimate the impact of nuclear T-bet and GATA3 expression on clinical outcomes, we conducted a Kaplan-Meier analysis to compare the OS between the negative-staining group and positive-staining group (including moderate and strong staining) in the patient cohort (Figure 2A). GATA3 expression was associated with reduced OS (log-rank, *p* = 0.000), while T-bet expression was not correlated with OS. The median OS observed in GATA3+ and GATA3- PTCL cases was 120 and 480 days, respectively. As shown in Table 3, a univariate survival analysis revealed that GATA3+ staining, presentation of B symptoms, elevated LDH level, ECOG > 2 and IPI > 3 were all predictors of worse prognosis (log-rank, *p* < 0.05). A multivariate Cox regression model including all the above factors showed that GATA3 expression (*p* = 0.004) and ECOG score (*p* = 0.000) were independent predictors of OS.

**Table 3:** Univariate and multivariate analyses for overall survival in 109 PTCL cases

Variable	*p*-Value for overall survival		HR	95% CI
	Univariate survival analysis	Multivariate survival analysis		
**Age (> 60)**	0.353			
**Gender (male)**	0.337			
**B symptom (present)**	0.007**	0.801		
**LDH (elevated)**	0.026*	0.100		
**ECOG (2–4)**	0.000**	0.000**	5.907	2.399–14.549
**Stage (III–IV)**	0.300			
**BM involvement (present)**	0.063			
**IPI score (3–5)**	0.001**	0.329		
**Ki-67 (> 70%)**	0.954			
**GATA3 (positive)**	0.000**	0.004**	2.651	1.372–5.121

To eliminate possible confounding effects of the heterogeneity of different pathological subgroups, we further analyzed the prognostic value of T-bet and GATA3 expression within subgroups. Due to the limited cohort size, we conducted stratified analyses in the PTCL-NOS and NKT groups only. In 60 PTCL-NOS cases, GATA3 expression identified a subset with reduced OS (log-rank, *p* = 0.002) (Figure 2B), while T-bet expression did not predict OS. In contrast, GATA3 failed to identify a specific prognostic subgroup among 18 NKT cases, while T-bet identified a subset of NKT with increased OS (log-rank, *p* = 0.004) (Figure 2C).

Of note, the survival curve for the GATA3+ group initially descended sharply, suggesting a rather short survival of some patients. In the Supplementary Materials (Supplementary Table S3), we listed the causes of death for all the patients living less than 20 days in the GATA3+ group. We also compared the rates of hemophagocytic syndrome between the 2 groups and found a higher incidence for the GATA3+ group (Fisher, *p* < 0.05; Table 4).

**Table 4:** Correlation of GATA3 expression with clinical characteristics

Clinical characteristics	GATA3		*P*-value
	Positive (cases)	Negative (cases)	
**B symptom**			
**Yes**	48	41	Fisher 0.006**
**No**	2	16	
**Hemophagocytic syndrome**			
**Yes**	9	2	Fisher 0.041*
**No**	42	56	
**CD68 expression**			
**Positive**	40	17	Pearson 0.000**
**Negative**	11	41	

### GATA3 expression is positively correlated with CD68 expression

Macrophages can be activated by T_H_2-associated cytokines and are linked with the T_H_2 response. GATA3 regulates T_H_2 differentiation by modulating T_H_2-associated cytokines. Therefore, we hypothesize that GATA3 may contribute to the activation of macrophages in tumor tissue. We used CD68 immunohistochemistry as a marker of infiltrated TAMs (see Supplementary Materials, Supplementary Figure S2). Large numbers of CD68+ cells were observed infiltrating tumor tissues and nearby stroma, whereas there were very few macrophages scattered throughout normal lymph node tissues. There was a positive correlation between GATA3 and CD68 expression (Pearson, *p* = 0.000). In addition, GATA3 expression was correlated with two clinical parameters, B symptom presentation (Pearson, *p* = 0.000; Table 4) and hemophagocytic syndrome incidence (Fisher, *p* = 0.041; Table 4).

### *GATA3* knockdown in T lymphoma cell lines leads to the decreased expression of several cytokines

**Figure 3:**
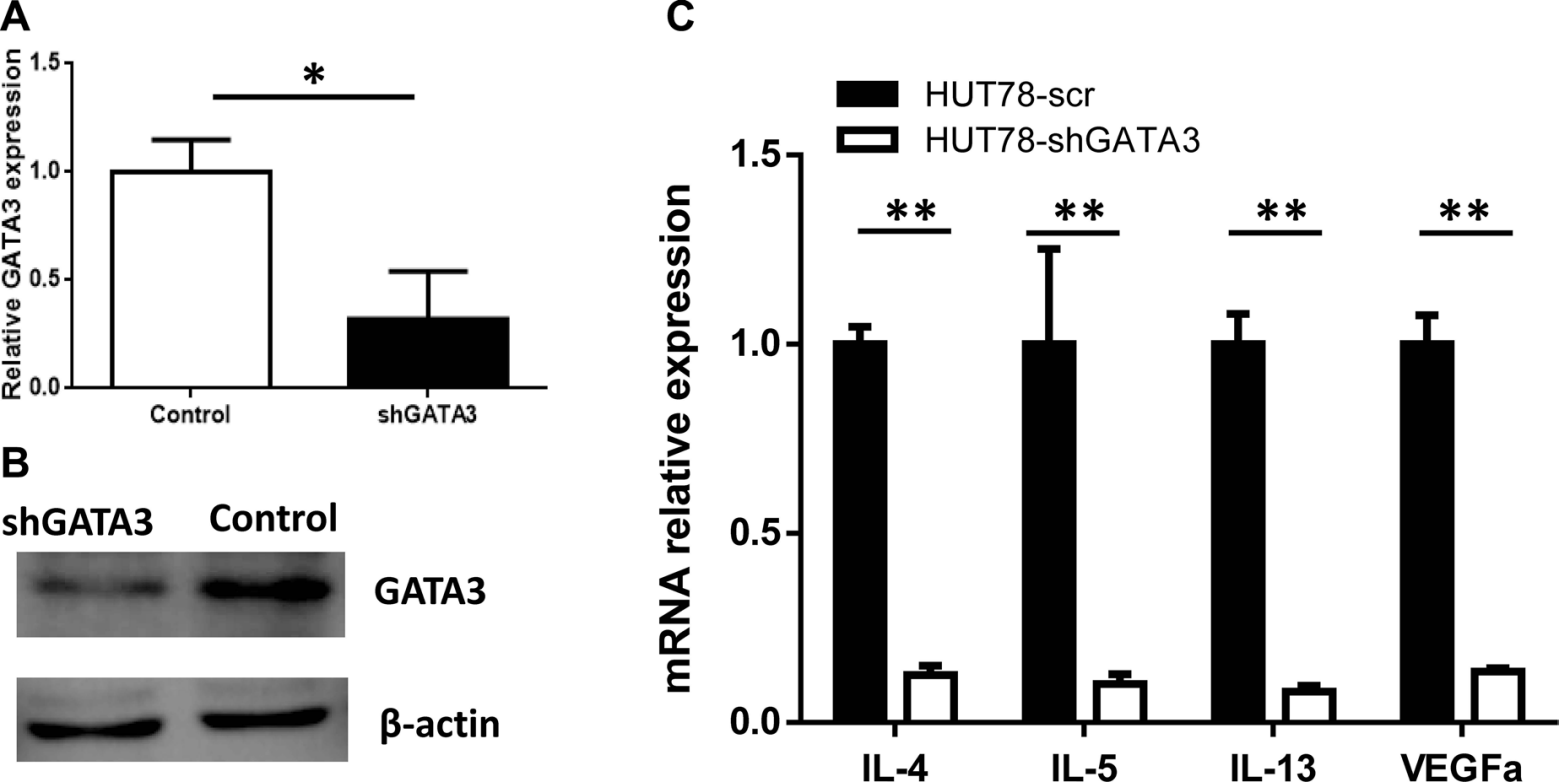
*GATA3* knockdown in T lymphoma cell lines leads to the decreased expression of T_H_2-associated cytokines (**A**) After *GATA3* shRNA lentiviral transfection, *GATA3* mRNA expression was decreased in Hut78 cells. (**B**) After GATA3 shRNA lentiviral transfection, GATA3 protein expression was decreased in Hut78 cells. (**C**) *GATA3* knockdown in Hut78 cells led to decreased expression of *IL-4, IL-5, IL-13*, and *VEGFA*.

To explore the possible mechanisms underlying the role of GATA3 in PTCL prognosis, we knocked down *GATA3* expression in the PTCL cell line, Hut78, using lentiviral transduction of an shRNA sequence. A cell line transfected with shLuciferase was also constructed as the control. Figure 3A and 3B, shows confirmation of *GATA3* knockdown at both the mRNA and protein levels. *GATA3* knockdown did not influence cell proliferation or cell viability, (Supplementary Figure S3B). However, in the *GATA3*-knockdown Hut78 cell line, expression of the T_H_2-associated cytokines *IL4*, *IL5*, and *IL13*, as well as *VEGFA* was decreased (Figure 3C).

### T lymphoma cells promote M2-type macrophage differentiation through a GATA3-dependent mechanism

**Figure 4:**
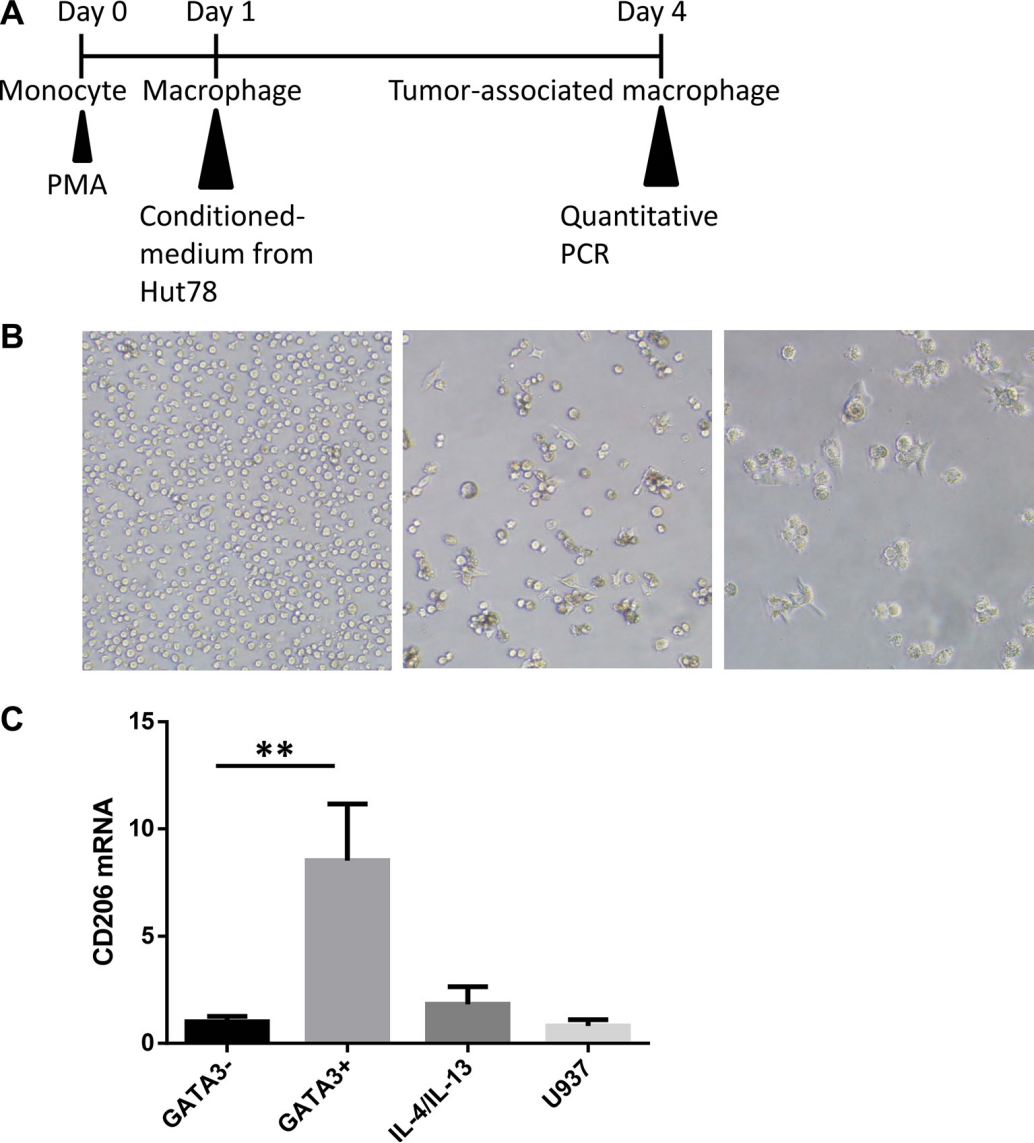
T lymphoma cells promote M2-type macrophage differentiation through a GATA3-dependent mechanism (**A**) The human monocyte cell line U937 was stimulated by phorbol 12-myristate 13-acetate (PMA) to induce differentiation into macrophages. The induced macrophages were divided into 4 groups and exposed to different media, including T lymphoma conditioned medium (1:1, v/v) or cytokine-containing media (20 ng/ml). After incubation for 72 h, the cells were collected and analyzed for CD206 expression. (**B**) U937 cells were incubated with PMA for 24 h. Cells adhered to the plate and began to show macrophage morphology. (**C**) Treatment of U937 cells with high GATA3 Hut78 cell-conditioned medium led to the highest expression of CD206, suggesting the highest percentage of M2 differentiation. Cells treated with the *GATA3*-knockdown Hut78 cell-conditioned medium displayed lower expression, while the IL-4/13-treated U937 cells had slightly higher expression.

Due to the correlation between GATA3 and CD68 expression, we designed *in vitro* studies to investigate the effect of GATA3 on macrophage polarization. As illustrated in Figure 4A, the human monocyte cell line U937 was stimulated by phorbol 12-myristate 13-acetate (PMA) to differentiate into macrophages. Twenty-four hours later, all the U937 cells had adhered to the dish and began to show macrophage morphology (Figure 4B). We then divided these macrophages into 4 groups exposed to various conditions, including T lymphoma conditioned medium (1:1, v/v) and various cytokines (20 ng/ml). After incubation for 72 h, we collected the cells and analyzed the expression of the M2-type macrophage differentiation marker CD206, as illustrated in Figure 4C. Treatment of U937 cells with GATA3-high Hut78 cell-conditioned medium led to the highest expression of *CD206*, suggesting the highest percentage of M2 differentiation. Treatment with the *GATA3* knockdown Hut78 cell-conditioned medium led to a lower expression of *CD206*, while treatment with IL- 4/13 led to a slightly higher expression of CD206.

## DISCUSSION

PTCL patients respond poorly to traditional chemotherapy regimens (such as CHOP) [10–12] and carry a dismal prognosis. Many researchers have turned to the newly-emerging bio-medicine field, in which several new drugs, such as monoclonal-antibodies (Brentuximab) [13], immune-modulators (Lenalidomide) [14], epigenetic modulators (Romidepsin) [15] and anti-metabolic drugs (Pralatrexate) [16] provide convincing results. This study screened several new prognostic biomarkers in PTCL tumor sections to catch a glimpse into the genetic and pathogenic mechanisms underlying PTCL.

Based on the level of GATA3 expression, PTCL cases can now be classified into at least 2 meaningful biological subgroups with distinct clinical outcomes. The GATA3+ subgroup was associated with a shorter OS. This conclusion is in agreement with two international analyses. Iqbal et al. [5] indicated that the group with high *GATA3* mRNA expression had better OS than the group with high *T-bet* expression. In a single center retrospective analysis, Wang et al. [9] performed a survival analysis and found a relationship between GATA3 expression and progression-free survival (PFS).

The mechanism underlying the role of GATA3 in tumorigenesis is not yet understood. Here we raise two hypotheses: 1) Because distinct cellular origins can cause vast differences in OS, GATA3+ tumor cells may indicate a cellular origin of T_H_2 lymphocytes, or 2) The transcription factor GATA3 may directly increase lymphoma cell malignancy through the up- or down-regulation of cytokine transcription or the activation of signal transduction pathways. Several pioneering studies in the field of B cell lymphoma provide supporting evidence for the first hypothesis, In 2000, Alizadeh et al. [17] published a gene expression profile study revealing that diffuse large B-cell lymphoma cases are comprised of two subgroups: activated B cell (ABC type) and germinal center B cell (GCB type). Subsequently, several independent research groups confirmed this breakthrough finding [18, 19]. Moreover, the Hans algorithm scoring system was established based on the IHC expression of several protein markers (Mum-1, Bcl-6, and CD10) [20–22]. Additional studies confirmed the different survival rates between these two groups, with the GCB type characterized by a superior survival rate and better response to Rituximab treatment as compared with the ABC type.

Naïve CD4-positive single lymphocytes can differentiate into different T helper cells, including T_H_1, T_H_2, and T_H_17, etc. [23]. There is evidence supporting that angioimmunoblastic T cell lymphoma may originate from T_FH_ cells [24]. HTLV virus-related adult T cell leukemia/lymphoma shares a similar immunophenotype with T_reg_ cells, with both displaying a high expression of FOXP3 [25]. However, the cellular origins of other PTCL subtypes remain unknown. Based on the phenomenon that the CD4-positive rate is higher than that for CD8 in PTCL-NOS [2], we deduce that the GATA3-high subgroup is of a T_H_2 origin, while the T-bet-high group is of a T_H_1 origin. We further explored this hypothesis using the clinical data of PTCL-NOS patients, such as the T_H_2-associated clinical symptoms. As IL-5, secreted by T_H_2 cells, is associated with eosinophilia syndrome [26], we compared the incidence of eosinophilia between the GATA3+ and GATA3- groups and failed to find a difference. This result is inconsistent with the findings of Wang et al, which found that the absolute eosinophilic count in the GATA3+ group was higher than in the GATA3- group. Wang's cohort included primarily PTCL-NOS cases with a small number of CTCL cases, while our cohort of 109 patients included various PTCL subtypes in addition to the 60 PTCL-NOS cases. The difference in the ratios of subgroup constituents may explain the different conclusions. In the future, we aim to validate the data in a larger sample.

Our correlation analysis (Table 4) may offer evidence supporting the second hypothesis of GATA3 regulation. When the survival curves were stratified by GATA3 expression, there was a more evident difference in OS during the earlier phase of follow-up than in the later phase. We hypothesized that the patients in the GATA3+ group were more susceptible to severe complications (such as severe B symptoms, HLH, etc.) when initiating a treatment regimen, leading to morbidity and mortality prior to the effects of chemotherapy.

There are several possible mechanisms that might explain this phenomenon. First, compared with the control group, the *GATA3*-knockdown PTCL cells had lower levels of T_H_2-associated cytokines (*IL4*, *IL5*, and *IL13*) and *VEGFA*. IL-4 and IL-13 are inflammatory cytokines and may elicit or aggravate the “cytokine storm,” leading to hemophagocytic syndrome in the bone marrow and elevated serum ferritin levels. Second, Geskin et al. found that cutaneous T-cell lymphoma (CTCL) cells were able to secrete IL-13 and express IL-13 receptors on their membranes. *In vitro* experiments indicated that IL-13 promotes CTCL tumor cell proliferation via auto-secretion and leads to a worse prognosis of CTCL patients [27]. Third, high GATA3 expression may directly up-regulate VEGFα secretion by tumor cells, thus promoting neovascularization in the tumor microenvironment and increasing the malignant behavior and metastatic ability of cancer cells.

The correlation analysis also found a positive relationship between GATA3 and CD68 expression, which is one of the markers of TAMs [28, 29]. Infiltration of TAMs in tumor tissue is correlated with poor prognosis, including in PTCL [8]. We induced macrophage differentiation through exposure to lymphoma-conditioned medium *in vitro*. There were less M2 type (CD206-positive) macrophages induced by the sh*GATA3* medium compared with the control medium. Thus, GATA3 expression may influence the constituents of TCL supernatants and indirectly change the ratio of induced M2 macrophages.

There are several factors that may be responsible for these key functions. Tumors secrete various cytokines, growth factors, chemokines, microRNAs and a few mRNAs [30]. In naïve T cells, GATA3 trans-activates the expression of T_H_2-associated cytokines (*IL4*, *IL5*, and *IL13*) [31] and indirectly down-regulates T_H_1-associated cytokines (*IFNG*) [32] to induce T cells towards T_H_2 differentiation. We deduced that GATA3 plays a similar role in the regulation of cytokines in T cell lymphoma. Our *in vitro* studies confirmed that the Hut78 cell line expressed lower levels of *IL4*, *IL5* and *IL13* mRNAs after *GATA3* knockdown. On the other hand, previous research on the tumor microenvironment implied that M2 type macrophages could be subdivided into M2a, M2b, and M2c types, with the M2a type induced by IL4/13 and playing roles in the T_H_2 response, Type 2 inflammatory response, and allergies [33]. CD206 is one of the M2a differentiation markers. Indeed, we detected a slight increase in CD206 expression in macrophages after *IL4* and *IL13* stimulation. We conclude that GATA3 promotes the differentiation of macrophages towards the M2 subtype by upregulating IL-4 and IL-13 secretion.

In contrast to Iqbal [5] et al, our study found a correlation between T-bet expression and PTCL prognosis. As there are differences in subtype constituents between the two studies, a selection bias may lead to an insignificant result. Moreover, our research focused on the protein expression of T-bet, while the study from Iqbal focused on the mRNA expression. It is not known whether there are post-translational modifications of T-bet that would lead to differential mRNA and protein expression. In addition, the incidence of PTCL in Asian populations is higher than in western populations [34]. Therefore, racial differences may influence the final conclusions of each of these studies.

Although our study did not find a relationship between T-bet and PTCL-NOS prognosis, it identified T-bet as a protective factor for NKT lymphoma patients, consistent with a study by Ren et al., which found that high T-bet expression was correlated with a favorable NKT lymphoma prognosis [35]. Several studies have offered a possible mechanism underlying this phenomenon. T-bet could up-regulate p53 expression in an NKT cell line, thus sensitizing the tumor cells' response to chemotherapy. Epstein-Barr Virus infection is commonly observed in NKT lymphoma patients. This virus can encode miR-BART20-5p, which inhibits T-bet translation, thus promoting resistance towards traditional cytotoxic agents in clinical patients [36]. In the future, we will expand our cohort of NKT lymphoma cases and validate the protective effects of T-bet in a larger number of samples in order to analyze the relationship between T-bet expression and EBV viral load.

## MATERIALS AND METHODS

### Patient samples and cell lines

A cohort of 109 PTCL patients visiting the Peking Union Medical College Hospital (PUMCH) between 2007 and 2013 was used in this study. The approval to review, analyze, and publish the data in this study was given by the PUMCH Research Ethics Board. Informed consent was provided according to the Declaration of Helsinki. PTCL cases were classified into different subgroups according to the 2010 WHO classification system. The most prevalent subtype was peripheral T-cell lymphoma-not otherwise specified (PTCL-NOS, *n* = 60, 55.0%), which was followed by NK/T-cell lymphoma (NKT, *n* = 18, 16.5%), angioimmunoblastic T cell lymphoma (AITL, *n* = 11, 10.1%), ALK^+^ anaplastic large cell lymphoma (ALCL, *n* = 4, 3.7%) and ALK^−^ ALCL (*n* = 4, 3.7%). Other subtypes included subcutaneous panniculitis-like T-cell lymphoma (SPTCL, *n* = 3, 2.8%), enteropathy-associated T cell lymphoma (EATL, *n* = 3, 2.8%), hepatosplenic T cell lymphoma (HSTCL, *n* = 2, 1.8%), γδT cell lymphoma (*n* = 2, 1.8%), and cutaneous T cell lymphoma (CTCL, *n* = 2, 1.8%). The PTCL cell lines and sources are shown in the Supplementary Material (Supplementary Table S1). Cell lines were maintained in RPMI1640 (China Infrastructure of Cell Line Resources) supplemented with 10% fetal bovine serum at 37%°C and 5% CO_2_.

### Immunohistochemistry

Formalin-fixed, paraffin-embedded tumor and normal lymph node tissues were stained with an anti-GATA3 antibody (dilution 1:200, Abcam, Ab98956), anti-T-bet antibody (dilution 1:50, Abcam, Ab150440), or anti-CD68 antibody (dilution 1:50, Dako, M071801). Antigen retrieval was used for the anti-GATA3, anti-T-bet and anti-CD68 antibodies via pressure-cooking (5 min). Appropriate negative (no primary antibody) and positive (normal tonsil or spleen sections) controls were used in parallel with each set of tumors studied. Scoring was independently performed by two pathologists, and inter-observer reproducibility was assessed based on the difference between the counts of each pathologist. Cases presenting nuclear staining in more than 10% of tumor cells were considered positive, grade 1 (pos+), whereas cases with strong nuclear staining in more than 30% of cells were considered positive, grade 2 (pos ++). For TAM quantification, cases with more than 20% CD68+ cells were considered positive. The percentage of CD68 cells was determined by the correlation of CD68 macrophage to the total number of non-neoplastic cells.

### Quantitative RT-PCR

Total RNA was isolated from cells using TRIzol reagent (Invitrogen, Grand Island, NY, USA). cDNA was synthesized using the RevertAid First Strand cDNA Synthesis Kit (Thermo Scientific, USA), and real-time PCR performed (in triplicate) using TransScript top green qPCR supermix (TransGen, Beijing, China). Human *GAPDH* expression was used as the endogenous control. All the primers used are listed in the Supplemental Material (Supplementary Table S2).

### Western blotting

Total protein was extracted from cell lines using M-PER Mammalian protein extraction reagent (Thermo Scientific, USA), supplemented with protease and phosphatase inhibitors. Western blotting was performed using a monoclonal primary mouse anti-human GATA3 antibody (Abcam, USA) and a β-ACTIN antibody as the loading control (Santa Cruz Biotechnology, USA).

### Lentiviral infection of lymphoma suspension cell lines

*GATA3* shRNA was cloned into pSIH-H1-copGFP shRNA cloning and expression lentivectors from SBI (System Biosciences, USA). The lentivirus was packaged by 293T cells and used to transfect T cell lymphoma cell lines. The cell lines stably expressing short hairpin RNAs (shRNAs) targeting *GATA3* were purified with GFP selection. The entire infection procedure is illustrated in Supplementary Figure S1.

### Statistical analysis

Patient clinical data were analyzed using the statistical software package SPSS (SPSS Inc., Cary, NJ, USA). Overall survival (OS) was estimated using the Kaplan-Meier method and 2-tailed log-rank test. GATA3 expression was dichotomized, and the Cox proportional hazards model was used to evaluate its ability to predict OS. Comparisons among groups were evaluated using a Student *t* test and *P* < 0.05 was determined to be statistically significant.

## CONCLUSIONS

In conclusion, this study found that the high expression of GATA3 is correlated with a poor prognosis in the general PTCL population as well as in PTCL-NOS, while high expression of T-bet is correlated with a favorable prognosis in NKTL. In addition, T lymphoma cells may promote the differentiation of M2-type macrophages through a GATA3-dependent mechanism. As a new DNAzyme targeting *GATA3* mRNA in T_H_2 cells entered Stage III clinical trials in treating asthma [37], These findings provide potential future therapeutic targets for peripheral T cell lymphoma.

## SUPPLEMENTARY MATERIALS FIGURES AND TABLE



## References

[1] Anderson JR, Armitage JO, Weisenburger DD (1998). Epidemiology of the non-Hodgkin's lymphomas: distributions of the major subtypes differ by geographic locations. Non-Hodgkin's Lymphoma Classification Project. Ann OncoL.

[2] Jaffe ES, Nicolae A, Pittaluga S (2013). Peripheral T-cell and NK-cell lymphomas in the WHO classification: pearls and pitfalls. Mod Pathol.

[3] William BM, Armitage JO (2013). International analysis of the frequency and outcomes of NK/T-cell lymphomas. Best Pract Res Cl Ha.

[4] Matsumoto Y, Horiike S, Ohshiro M, Yamamoto M, Sasaki N, Tsutsumi Y, Kobayashi T, Shimizu D, Uchiyama H, Kuroda J, Nomura K, Shimazaki C, Taniwaki M (2010). Expression of Master Regulators of Helper T-Cell Differentiation in Peripheral T-Cell Lymphoma, Not Otherwise Specified, by Immunohistochemical Analysis. Am J Clin Pathol.

[5] Iqbal J, Wright G, Wang C, Rosenwald A, Gascoyne RD, Weisenburger DD, Greiner TC, Smith L, Guo S, Wilcox RA, Teh BT, Lim ST, Tan SY (2014). Gene expression signatures delineate biological and prognostic subgroups in peripheral T-cell lymphoma. Blood.

[6] Pollard JW (2004). Tumour-educated macrophages promote tumour progression and metastasis. Nat Rev Cancer.

[7] Weigert A, Sekar D, Brune B (2009). Tumor-associated macrophages as targets for tumor immunotherapy. Immunotherapy-Uk.

[8] Zhang W, Wang L, Zhou D, Cui Q, Zhao D, Wu Y (2011). Expression of tumor-associated macrophages and vascular endothelial growth factor correlates with poor prognosis of peripheral T-cell lymphoma, not otherwise specified. Leuk Lymphoma.

[9] Wang T, Feldman AL, Wada DA, Lu Y, Polk A, Briski R, Ristow K, Habermann TM, Thomas D, Ziesmer SC, Wellik LE, Lanigan TM, Witzig TE (2014). GATA-3 expression identifies a high-risk subset of PTCL, NOS with distinct molecular and clinical features. Blood.

[10] Petrich AM, Rosen ST (2013). Peripheral T-cell lymphoma: new therapeutic strategies. Oncology (Williston Park).

[11] Shibata Y, Hara T, Kasahara S, Yamada T, Sawada M, Mabuchi R, Matsumoto T, Nakamura N, Nakamura H, Ninomiya S, Kitagawa J, Kanemura N, Kito Y (2015). CHOP or THP-COP regimens in the treatment of newly diagnosed peripheral T-cell lymphoma, not otherwise specified: a comparison of doxorubicin and pirarubicin. Hematol Oncol.

[12] Zelenetz AD, Gordon LI, Wierda WG, Abramson JS, Advani RH, Andreadis CB, Bartlett N, Byrd JC, Czuczman MS, Fayad LE, Fisher RI, Glenn MJ, Harris NL (2014). Non-Hodgkin's lymphomas, version 4. 2014. J Natl Compr Canc Netw.

[13] Horwitz SM, Advani RH, Bartlett NL, Jacobsen ED, Sharman JP, O'Connor OA, Siddiqi T, Kennedy DA, Oki Y (2014). Objective responses in relapsed T-cell lymphomas with single-agent brentuximab vedotin. Blood.

[14] Toumishey E, Prasad A, Dueck G, Chua N, Finch D, Johnston J, van der Jagt R, Stewart D, White D, Belch A, Reiman T (2015). Final report of a phase 2 clinical trial of lenalidomide monotherapy for patients with T-cell lymphoma. Cancer-Am Cancer Soc.

[15] Coiffier B, Pro B, Prince HM, Foss F, Sokol L, Greenwood M, Caballero D, Borchmann P, Morschhauser F, Wilhelm M, Pinter-Brown L, Padmanabhan S, Shustov A (2012). Results from a pivotal, open-label, phase II study of romidepsin in relapsed or refractory peripheral T-cell lymphoma after prior systemic therapy. J Clin Oncol.

[16] O'Connor OA, Pro B, Pinter-Brown L, Bartlett N, Popplewell L, Coiffier B, Lechowicz MJ, Savage KJ, Shustov AR, Gisselbrecht C, Jacobsen E, Zinzani PL, Furman R (2011). Pralatrexate in patients with relapsed or refractory peripheral T-cell lymphoma: results from the pivotal PROPEL study. J Clin Oncol.

[17] Alizadeh AA, Eisen MB, Davis RE, Ma C, Lossos IS, Rosenwald A, Boldrick JC, Sabet H, Tran T, Yu X, Powell JI, Yang L, Marti GE (2000). Distinct types of diffuse large B-cell lymphoma identified by gene expression profiling. Nature.

[18] Blenk S, Engelmann J, Weniger M, Schultz J, Dittrich M, Rosenwald A, Muller-Hermelink HK, Muller T, Dandekar T (2007). Germinal center B cell-like (GCB) and activated B cell-like (ABC) type of diffuse large B cell lymphoma (DLBCL): analysis of molecular predictors, signatures, cell cycle state and patient survival. Cancer Inform.

[19] Rosenwald A, Wright G, Chan WC, Connors JM, Campo E, Fisher RI, Gascoyne RD, Muller-Hermelink HK, Smeland EB, Giltnane JM, Hurt EM, Zhao H, Averett L (2002). The use of molecular profiling to predict survival after chemotherapy for diffuse large-B-cell lymphoma. N Engl J Med.

[20] Muris JJ, Meijer CJ, Vos W, van Krieken JH, Jiwa NM, Ossenkoppele GJ, Oudejans JJ (2006). Immunohistochemical profiling based on Bcl-2, CD10 and MUM1 expression improves risk stratification in patients with primary nodal diffuse large B cell lymphoma. J Pathol.

[21] Hans CP, Weisenburger DD, Greiner TC, Gascoyne RD, Delabie J, Ott G, Muller-Hermelink HK, Campo E, Braziel RM, Jaffe ES, Pan Z, Farinha P, Smith LM (2004). Confirmation of the molecular classification of diffuse large B-cell lymphoma by immunohistochemistry using a tissue microarray. Blood.

[22] Choi WW, Weisenburger DD, Greiner TC, Piris MA, Banham AH, Delabie J, Braziel RM, Geng H, Iqbal J, Lenz G, Vose JM, Hans CP, Fu K (2009). A new immunostain algorithm classifies diffuse large B-cell lymphoma into molecular subtypes with high accuracy. Clin Cancer Res.

[23] Grogan JL, Locksley RM (2002). T helper cell differentiation: on again, off again. Curr Opin Immunol.

[24] de Leval L, Rickman DS, Thielen C, Reynies A, Huang YL, Delsol G, Lamant L, Leroy K, Briere J, Molina T, Berger F, Gisselbrecht C, Xerri L, Gaulard P (2007). The gene expression profile of nodal peripheral T-cell lymphoma demonstrates a molecular link between angioimmunoblastic T-cell lymphoma (AITL) and follicular helper T (TFH) cells. Blood.

[25] Roncador G, Garcia JF, Garcia JF, Maestre L, Lucas E, Menarguez J, Ohshima K, Nakamura S, Banham AH, Piris MA (2005). FOXP3, a selective marker for a subset of adult T-cell leukaemia/lymphoma. Leukemia.

[26] Simon HU, Plotz SG, Dummer R, Blaser K (1999). Abnormal clones of T cells producing interleukin-5 in idiopathic eosinophilia. N Engl J Med.

[27] Geskin LJ, Viragova S, Stolz DB, Fuschiotti P (2015). Interleukin-13 is overexpressed in cutaneous T-cell lymphoma cells and regulates their proliferation. Blood.

[28] Steidl C, Lee T, Shah SP, Farinha P, Han G, Nayar T, Delaney A, Jones SJ, Iqbal J, Weisenburger DD, Bast MA, Rosenwald A, Muller-Hermelink HK (2010). Tumor-associated macrophages and survival in classic Hodgkin's lymphoma. N Engl J Med.

[29] Steidl C, Farinha P, Gascoyne RD (2011). Macrophages predict treatment outcome in Hodgkin's lymphoma. Haematologica.

[30] Wang W, Luo YP (2015). MicroRNAs in breast cancer: oncogene and tumor suppressors with clinical potential. J Zhejiang Univ Sci B.

[31] Ansel KM, Djuretic I, Tanasa B, Rao A (2006). Regulation of Th2 differentiation and Il4 locus accessibility. Annu Rev Immunol.

[32] Ouyang W, Ranganath SH, Weindel K, Bhattacharya D, Murphy TL, Sha WC, Murphy KM (1998). Inhibition of Th1 development mediated by GATA-3 through an IL-4-independent mechanism. Immunity.

[33] Martinez FO, Gordon S (2014). The M1 and M2 paradigm of macrophage activation: time for reassessment. F1000Prime Rep.

[34] Tang T, Tay K, Quek R, Tao M, Tan SY, Tan L, Lim ST (2010). Peripheral T-cell lymphoma: review and updates of current management strategies. Adv Hematol.

[35] Ren YL, Nong L, Zhang S, Zhao J, Zhang XM, Li T (2012). Analysis of 142 Northern Chinese Patients With Peripheral T/NK-Cell Lymphomas: Subtype Distribution, Clinicopathologic Features, and Prognosis. Am J Clin Pathol.

[36] Lin TC, Liu TY, Hsu SM, Lin CW (2013). Epstein-Barr virus-encoded miR-BART20–5p inhibits T-bet translation with secondary suppression of p53 in invasive nasal NK/T-cell lymphoma. Am J Pathol.

[37] Bochner BS, Schleimer RP (2015). Out of the orphanage and into the clinic—therapeutic targeting of GATA3. N Engl J Med.

