# Supplementary material for: GATA3 expression correlates with poor prognosis and tumor-associated macrophage infiltration in peripheral T cell lymphoma
Source: Oncotarget. 2016 Aug 29;7(40):65284–94. doi: 10.18632/oncotarget.11673 (PMC5323155; doi:10.18632/oncotarget.11673)
Supplement: Supplementary file 1 [file oncotarget-07-65284-s001.pdf]

GATA3 expression correlates with poor prognosis and tumor-associated macrophage infiltration in peripheral T cell lymphoma

Supplementary Materials

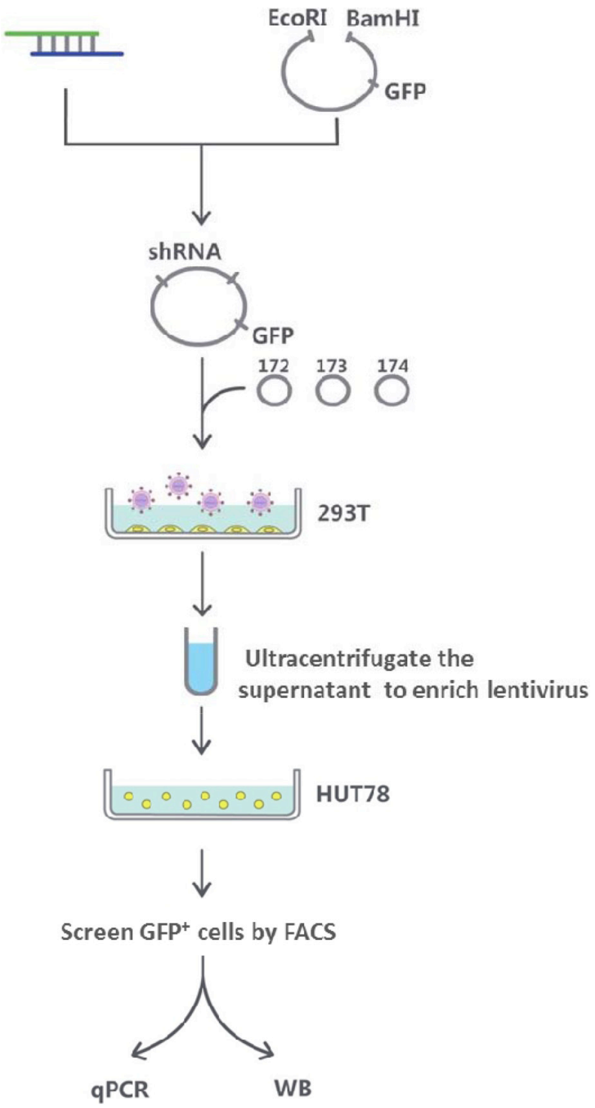

Supplementary Figure S1: The procedure of constructing GATA3-knockdown Hut78 cell line. GATA3 shRNA was cloned into pSIH-H1-copGFP shRNA cloning and expression lentivectors from SBI (System Biosciences, USA). Lentivirus was packaged by 293T cells and used to transfect T cell lymphoma cell lines. The cell lines stably expressing short hairpin RNAs (shRNAs) targeting GATA-3 were purified by GFP selection.

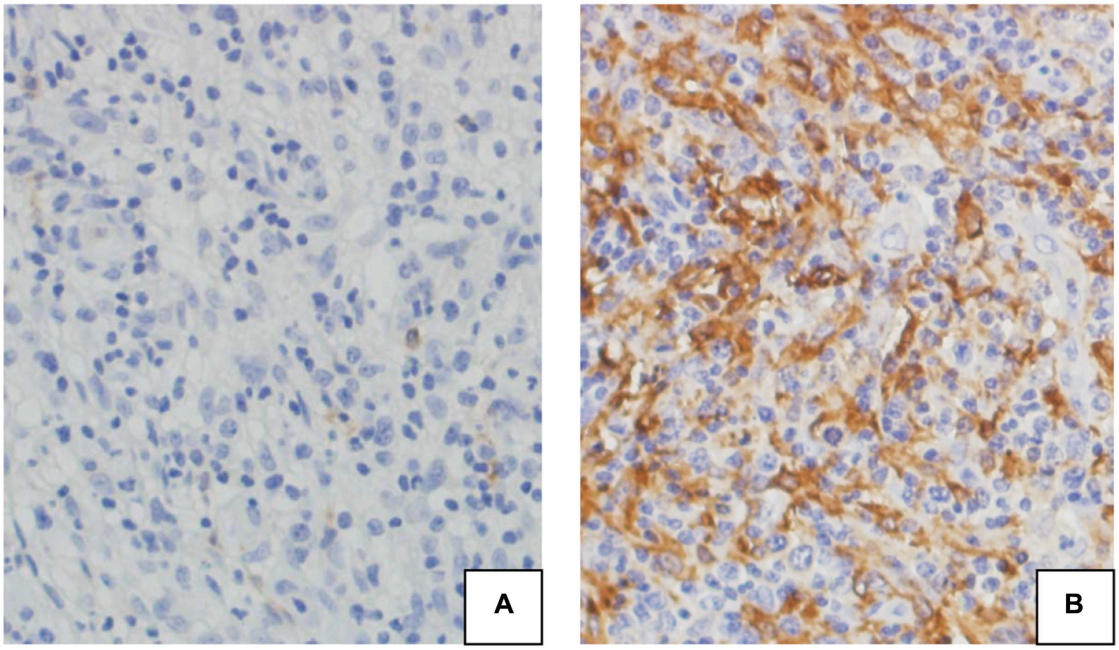

Supplementary Figure S2: The expression of CD68 in different PTCL tumor tissues. (**A**) Negative staining. (**B**) Positive staining.

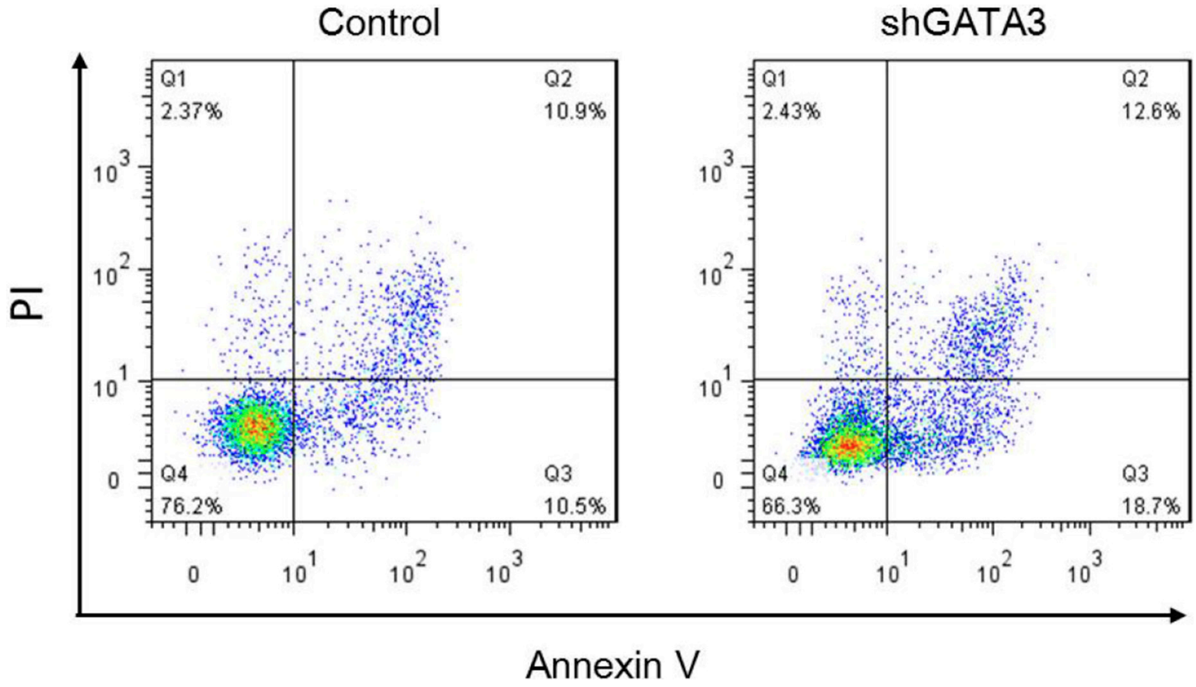

Supplementary Figure S3: Apoptosis assay by flow cytometry. Cell viability was measured by flow cytometry using Annexin V and PI staining. We failed to observe prominent differences between GATA3-knockdown Hut78 and control group.

Supplementary Table S1: Cell line information and sources

Cell line	Histology	Cell source	Culture property
**HUT78**	CTCL (Sezary Syndrome)	China Infrastructure of Cell Line Resources	Suspension growth, partially in aggregates
**Jurkat**	ATLL (HTLV-1-)	China Infrastructure of Cell Line Resources	Suspension growth, in aggregates
**Karpass299**	PTCL (ALCL, ALK+)	Generous gift from the First Affiliated Hospital of Zhengzhou University	Suspension growth
**U937**	Human histocytic leukemia	China Infrastructure of Cell Line Resources	Suspension growth
**293T**	Human renal epithelial cell transfected with adenovirus E1A	China Infrastructure of Cell Line Resources	Adherent growth

Supplementary Table S2: Primer sequences

Gene	Primer sequences 5′ → 3′
GATA3	Forward ACACTCTGGAGGAGGAATGCCAAT<br>Reverse TTCGGTTTCTGGTCTGGATGCCTT
IL-4	Forward GGGCTTGAATTCCTGTCCTGT
	Reverse TCGTCTTTAGCCTTTCCAAGAAGT
IL-5	Forward AGCTGCCTACGTGTATGCCA
	Reverse GCAGTGCCAAGGTCTCTTTCA
IL-13	Forward TCTGCAATGGCAGCATGGTA
	Reverse GCATCCTCTGGGTCTTCTCG
VEGFa	Forward AGGGCAGAATCATCACGAAGT
	Reverse AGGGTCTCGATTGGATGGCA
CD206	Forward CGCTACTAGGCAATGCCAATG
	Reverse GCAATCTGCGTACCACTTGTTTT
GAPDH	Forward GAAGGTGAAGGTCGGAGTC
	Reverse GAAGATGGTGATGGGATTTC

Supplementary Table S3: Death causes of 16 patients with OS < 20 days

Death cause	Case number
Hemophagocytic syndrome	5
Septic shock, multiple organ dysfunction syndrome	6
Gastrointestinal bleeding	2
CNS involvement	1
DIC, major bleeding	1
Multiple organ dysfunction syndrome	1
